# A Comprehensive Metabolomic and Microbial Analysis Following Dietary Amino Acid Reduction in Mice

**DOI:** 10.3390/metabo14120706

**Published:** 2024-12-14

**Authors:** Raghad Khalid Al-Ishaq, Carmen R. Ferrara, Nisha Stephan, Jan Krumsiek, Karsten Suhre, David C. Montrose

**Affiliations:** 1 Bioinformatics Core, Weill Cornell Medicine-Qatar, Cornell University, Education City, Doha 24144, Qatar; 2 Department of Pathology, Renaissance School of Medicine, Stony Brook University, MART Building, 9M-0816, Lauterbur Dr., Stony Brook, NY 11794, USA; 3 Department of Physiology and Biophysics, Weill Cornell Medical College, New York, NY 10065, USA; 4 Sandra and Edward Meyer Cancer Center, New York, NY 11215, USA; 5 Caryl and Israel Englander Institute for Precision Medicine, New York, NY 10021, USA; 6 Stony Brook Cancer Center, Stony Brook, NY 11794, USA

**Keywords:** metabolomics, diet, protein, bacteria, 16S, nutrition, amino acids

## Abstract

**Introduction:** Nutritional metabolomics provides a comprehensive overview of the biochemical processes that are induced by dietary intake through the measurement of metabolite profiles in biological samples. However, there is a lack of deep phenotypic analysis that shows how dietary interventions influence the metabolic state across multiple physiologic sites. Dietary amino acids have emerged as important nutrients for physiology and pathophysiology given their ability to impact cell metabolism. **Methods:** The aim of the current study is to evaluate the effect of modulating amino acids in diet on the metabolome and microbiome of mice. Here, we report a comprehensive metabolite profiling across serum, liver, and feces, in addition to gut microbial analyses, following a reduction in either total dietary protein or diet-derived non-essential amino acids in mice. **Results:** We observed both distinct and overlapping patterns in the metabolic profile changes across the three sample types, with the strongest signals observed in liver and serum. Although amino acids and related molecules were the most commonly and strongly altered group of metabolites, additional small molecule changes included those related to glycolysis and the tricarboxylic acid cycle. Microbial profiling of feces showed significant differences in the abundance of select species across groups of mice. **Conclusions:** Our results demonstrate how changes in dietary amino acids influence the metabolic profiles across organ systems and the utility of metabolomic profiling for assessing diet-induced alterations in metabolism.

## 1. Introduction

Diet contributes to whole body metabolism, given the importance of diet-derived nutrients in mediating intracellular signaling and organ physiology [1,2,3,4]. As such, dietary factors play a key role in mediating both organism homeostasis and disease pathogenesis, including cancer [5,6,7,8,9]. For example, restriction or supplementation of key macronutrients including carbohydrates or amino acids have shown promise, at least in the preclinical setting, for suppressing tumorigenesis and enhancing response to anti-cancer therapy [5,6,7,8,9]. These anti-neoplastic effects result, in large part, from depriving cancer cells of critical mediators used to support intracellular metabolism-processes that drive proliferation, metastatic dissemination and resistance to therapy [5,6,7,8,9]. The role of diet in modulating these pathways could be mediated directly by supplying metabolites to cells. Further, given the impact of diet on gut microbiota, altering their populations or metabolism could additionally impact normal or neoplastic host cells [10,11,12]. Importantly, altering diet to prevent or slow the progression of cancer, as well as enhance cancer therapy, is gaining widespread interest, highlighting the importance of better understanding the impact of such an approach on whole body metabolism.

Over the last decade, metabolomics has come to the forefront as an informative omics approach, providing insight into metabolic reactions in multiple biologic matrices, including tissues, cells, fluids, and feces [13,14,15]. In fact, metabolite profiling has been critical for identifying key metabolic pathways for driving disease [16,17,18,19]. Equally as important has been the development of computational approaches to both accurately assess meaningful changes in the abundance of small molecules and integrate metabolite changes with other omics platforms [20,21,22,23]. For example, statistical analysis tools dedicated to metabolomics, such as MetaboAnalyst [22] and Maplet [20], allow for the integrated analysis of large and complex datasets from mouse and human studies, including multiple tissues, platforms and omics modalities [16,19]. Given the influence of dietary nutrients on the metabolism of organs and cells, metabolomics and its associated computational analyses provide an especially useful readout of the impact of diet. In fact, several studies have shown the utility of so-called nutritional metabolomics for providing critical insights into the interplay between diet and biochemical pathways, and their impact on disease pathogenesis, as well as assessing how dietary changes affect circulating or gut metabolites [24,25]. However, a better understanding of the impact of dietary protein/amino acids, critical mediators of physiology and pathophysiology, remains to be established, along with the optimal methodology for analyzing its metabolic effects. The aim of this study was to determine the physiologic response to reducing dietary amino acids in multiple organ systems through a multi-omics approach. Such information will be critical if modifying amino acid intake or other nutrients is to be implemented as a therapeutic approach in patients.

## 2. Materials and Methods

### 2.1. Diets and Mouse Treatments

Male 8-week old A/J mice (The Jackson Laboratory, Bar Harbor, ME, USA) were fed an AIN-93G purified diet (Research Diets, Brunswick, NJ, USA) while bedding swapping was performed twice per week for two weeks to homogenize microbiota. Following this period, mice were evenly divided into three groups, singly housed and pair-fed either AIN-93G (control), AIN-93G with 50% reduced casein (Reduced protein diet) or AIN-93G with 50% reduced casein and essential amino acids added back to the levels found in the casein of the control diet (Reduced NEAA diet) (n = 4–6/group) (Table 1). Soybean oil was added to make the experimental diets isocaloric compared to the control diet (Table 1). Pair-feeding was accomplished by measuring the amount of diet consumed by the control group over a 24 h period; then, the amount of the two experimental diets (by weight) was provided to the other groups of mice on a daily basis. Mice were fed each of these diets for 2 weeks; then, feces were collected and snap-frozen in liquid nitrogen and stored at −80 °C until analysis. Mice were then euthanized, and blood was collected by cardiac puncture to generate serum, which was stored at −20 °C prior to analysis. Liver tissue was harvested and snap-frozen, then stored at −80 °C until analysis.

### 2.2. Metabolomics

Metabolite profiling of feces and serum was carried out at the Weill Cornell Medical College Proteomics and Metabolomics Core Facility, as previously described [26]. Briefly, metabolites were extracted from feces (after drying down) and liver by bead-beating in cold 80% methanol, then held for 4 h at 80 °C. Samples were centrifuged and supernatants transferred for further processing. Serum metabolites were extracted by vortexing in cold 80% methanol, then held for 4 h at −20 °C, followed by centrifugation. Supernatants from all sample types were dried by vacuum centrifugation and resuspended in mobile phase. LC/MS analysis was performed on a Vanquish UPLC system coupled to a Q Exactive Orbitrap mass spectrometer (Thermo Scientific, Waltham, MA, USA) operating in polarity-switching mode. A Sequant ZIC-HILIC column (2.1 mm i.d. × 150 mm, Merck, Rahway, NJ, USA) was used to separate metabolites. For mobile A, 100% acetonitrile was used, and 0.1% NH_4_OH/20 mmol/L CH_3_COONH_4_ in water was used for mobile B. The gradient ran from 85% to 30% A in 20 min followed by a wash with 30% A and re-equilibration at 85% A. Metabolites were identified based on exact masses within 5 ppm and standard retention times. Relative metabolite quantification was performed based on peak area for each metabolite and abundance was normalized to weight for feces and liver and volume for serum.

### 2.3. 16S rRNA Profiling

Feces were collected from individual mice and snap-frozen in liquid nitrogen. Samples were shipped to Molecular Research (Shallowater, TX, USA) for 16S rRNA profiling, as previously described [27,28]. Briefly, DNA extraction was carried out using the Powersoil DNA Kit (Qiagen, Hilden, Germany) per the manufacturer’s instructions. The 16S rRNA gene V4 variable region was then amplified by PCR primers 515/806 with barcode on the forward primer used in a 30-cycle PCR using the HotStarTaq Plus Master Mix Kit (Qiagen). The amplified DNA was purified using Ampure XP beads for the construction of sequencing libraries. DNA sequencing was performed on an Illumina HiSeq system (Illumina). Sequence data were processed by using the pipeline of MR DNA. Briefly, sequences were joined after depletion of barcodes and short sequences or sequences with ambiguous base calls. Sequences were denoised, operational taxonomic units (OTUs) were generated, and chimeras were removed. OTUs were defined by clustering at 3% divergence. OTUs were taxonomically classified by using BLASTn against a database derived from RDPII and NCBI (www.ncbi.nlm.nih.gov, http://rdp.cme.msu.edu).

### 2.4. Data Processing and Statistical Analysis

All data were processed and analyzed using the “Maplet” package in R [20]. Metabolite abundance data from the three sample types (liver, serum and feces) and the microbiome data were normalized using quotient normalization and data were log-transformed. Data with >20% missing values were excluded from the data set to prevent artifacts during Pearson correlation analysis. The remaining missing values were imputed to the minimum concentration determined for a given metabolite/microbial species. After processing, the data set comprised 437 metabolites (liver = 161; serum = 158; feces = 118) and 64 microbial species. ANOVA and pairwise comparisons were performed on all 437 metabolite traits from the three tissues and the 64 microbial species. A Bonferroni level of significance of 9.98 × 10^−5^ (0.05/(437 + 64)) was used.

## 3. Results

### 3.1. Reducing Dietary Amino Acids Induces a Shift in the Metabolome of Liver, Serum and Feces

**Figure 1:**
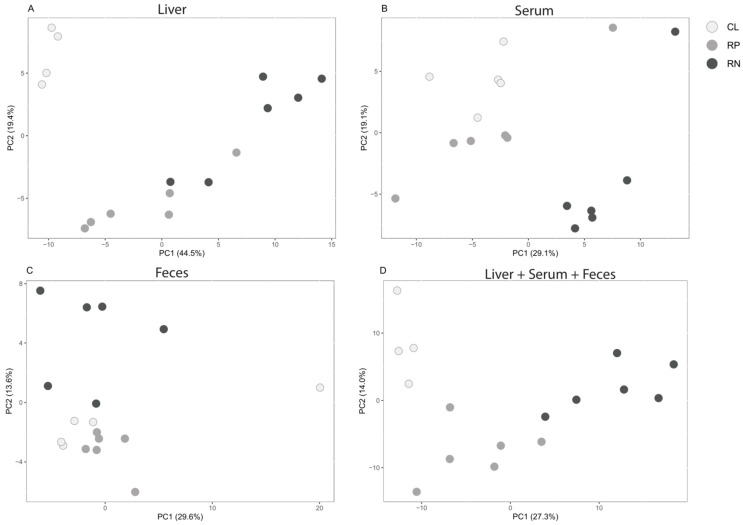
Global metabolite shifts occur in response to modifying dietary amino acids. Principal component analysis (PCA) was conducted on the metabolite profiles of livers (**A**), serum (**B**), feces (**C**) or all three matrices combined (**D**) from mice fed control (CL), reduced protein (RP) or reduced NEAA (RN) diets for two weeks.

Mice were pair-fed either an AIN-93G purified diet to serve as the control group (CL), a purified diet in which protein was reduced by 50% (RP) or a purified diet in which non-essential amino acids (NEAAs) were reduced by 50% (RN), for two weeks (Table 1). Measurements of calorie consumption and body weight revealed no major differences across the groups during the two-week feeding period (Supplementary Figure S1). At the end of the dietary intervention, liver tissue, serum and feces were collected for subsequent metabolomic profiling. Following processing of data (see Section 2 Materials and Methods), a total of 204 unique metabolites were available for comparative analysis, with 161, 158 and 118 metabolites identified in the liver, serum and feces, respectively (Supplementary Table S1). Principal component analysis (PCA) revealed equivalently strong but somewhat distinct shifts for each of the experimental diets in the livers, compared to those mice fed the CL diet (Figure 1A). Serum samples also exhibited alterations in metabolite profiles induced by the modified diets, with a greater shift in those mice fed the RN diet (Figure 1B). Analysis of feces revealed that only the RN diet was capable of inducing an observable shift (Figure 1C). Lastly, we integrated the metabolomic profiles of all three sample types from mice fed either of the three diets and found that diet type appeared to be a major driver of metabolite abundance regardless of physiologic site (Figure 1D).

### 3.2. The Liver Metabolome Is Most Strongly Effected by Modifying Amino Acid Intake

**Figure 2:**
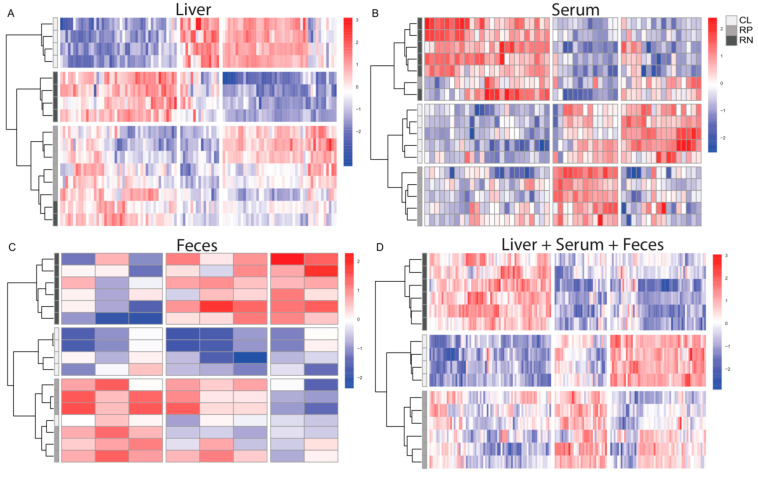
Reducing protein or non-essential amino acids in diet induces significant changes in the levels of metabolites in the liver, serum and feces. Small molecules that were significantly changed (*p* adj < 0.05) in abundance in the livers (**A**), serum (**B**), feces (**C**) or all three matrices combined (**D**) from mice fed a reduced protein (RP) or reduced NEAA (RN) diet compared to mice given a control diet (CL) are shown as heat maps. Red color indicates increased abundance; blue color indicates decreased abundance.

Hierarchical clustering of metabolite changes revealed general segregation of diet types for each of the biological matrices (Figure 2A–C). Interestingly, perfect separation occurred when all sample types were combined, suggesting that diet is a major driver of metabolite changes regardless of biological matrix (Figure 2D). Examination of the direction of change across diets and sample types shows numerous significantly changed metabolites that were either higher or lower in abundance upon reducing total protein or NEAAs, compared to CL diet, in each of the matrices (Figure 2 and Supplementary Table S1). Overall, the greatest number of changes occurred in livers upon feeding either experimental diet, compared to other sample types (Figure 2 and Supplementary Table S1). Additionally, more robust and distinct changes occurred in the RN group for the majority of sample types, compared to the RP group, and when sample types were combined (Figure 2 and Supplementary Table S1). Given the marked changes observed in livers and the importance of dietary amino acids for supporting cell metabolism, we more closely examined two major metabolic pathways, glycolysis and the tricarboxylic acid (TCA) cycle. These analyses revealed reduced levels of most glycolytic intermediates, as well as lactate, in mice fed either experimental diet, with a more pronounced effect in the RN group (Supplementary Figure S2A). Measurements of TCA cycle intermediates showed that although citrate was increased in both the RP and RN groups, downstream metabolites were largely decreased (Supplementary Figure S2B). These data suggest that reducing amino acid intake alters the metabolic landscape of the liver.

### 3.3. Changes in the Physiologic Levels of Amino Acids Do Not Fully Reflect the Reduction in Dietary Source

**Figure 3:**
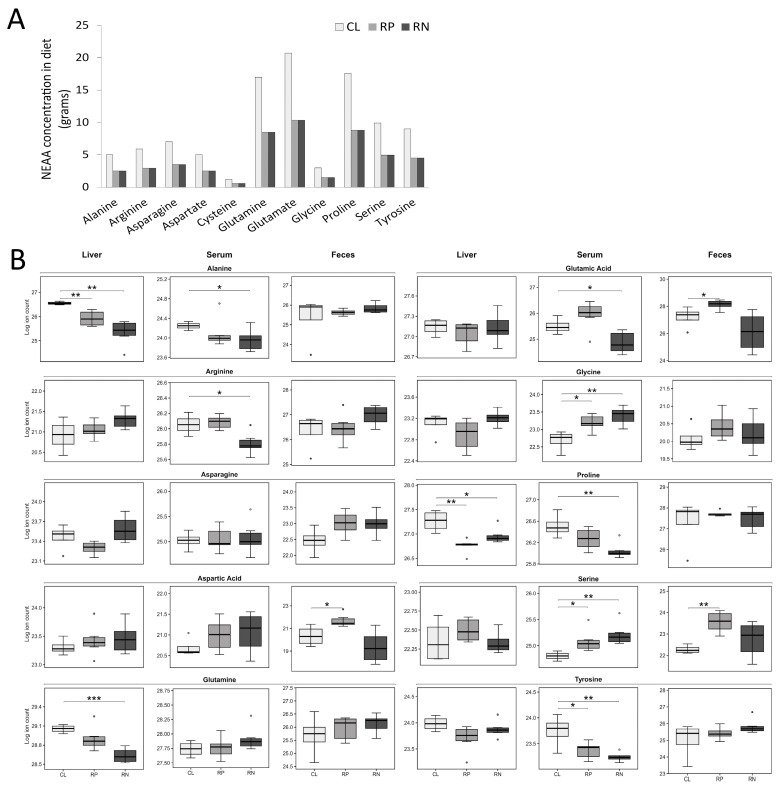
Non-essential amino acid levels change in multiple biological matrices following consumption of amino acid-modified diets. (**A**) The abundance of non-essential amino acids in each of the study diets is shown. (**B**) The relative levels of non-essential amino acids in the liver, serum and feces from mice fed a reduced protein (RP) or reduced NEAA (RN) diet compared to mice given a control diet (CL) are shown (* *p* < 0.05; ** *p* < 0.01; *** *p* < 0.001).

**Figure 4:**
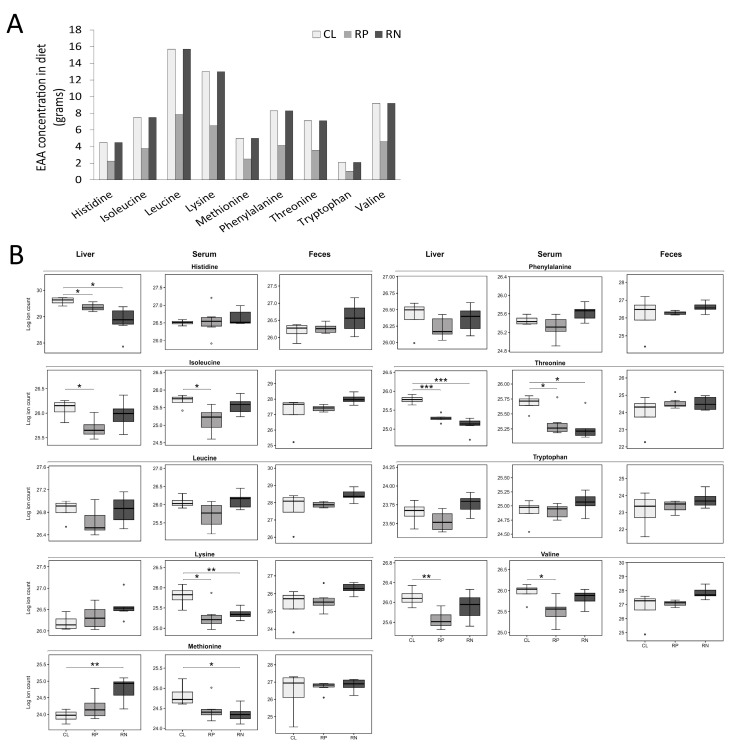
Essential amino acid levels change in multiple biological matrices following consumption of amino acid-modified diets. (**A**) The abundance of essential amino acids in each of the study diets is shown. (**B**) The relative levels of essential amino acids in the liver, serum and feces from mice fed a reduced protein (RP) or reduced NEAA (RN) diet compared to mice given a control diet (CL) are shown (* *p* < 0.05; ** *p* < 0.01; *** *p* < 0.001).

Because the dietary alterations used in this study focused on protein and amino acids, the relative abundance of amino acids in each of the three sample types was selectively examined. We first depicted the amount of each essential and NEAA contained in the experimental diets based on their respective levels found in casein to provide a basis for comparison to amino acid abundance in the biological samples (Figure 3A and Figure 4A). Next, pairwise comparisons were made between the three experimental diets to determine how altering protein/amino acid abundance impacted amino acid levels in liver, serum and feces of mice. Serum samples revealed that while tyrosine was the only significantly decreased NEAA in mice fed the RP diet, an additional 4 NEAAs were lower in mice fed the RN diet (Figure 3B and Supplementary Table S1). Interestingly, glycine and serine both increased in the serum of mice fed either diet (Figure 3B and Supplementary Table S1). Examination of EAA levels in serum showed a nearly equivalent decrease in mice fed either experimental diet, including overlapping decreases in lysine and threonine (Figure 4B; Supplementary Table S1). Analysis of liver samples revealed a similar decrease in the NEAAs proline and alanine and the EAAs threonine and histidine in both experimental diets, with additional distinct reductions in other AAs across diets (Figure 3B, Figure 4B and Supplementary Table S1). Fecal analysis of amino acid levels showed that only serine changed in abundance, which increased upon feeding the RP diet, with a similar but non-significant trend in feces from mice fed the RN diet (Figure 3B and Supplementary Table S1).

### 3.4. Reducing Dietary Protein or Amino Acids Alters the Abundance of Select Bacterial Species

**Figure 5:**
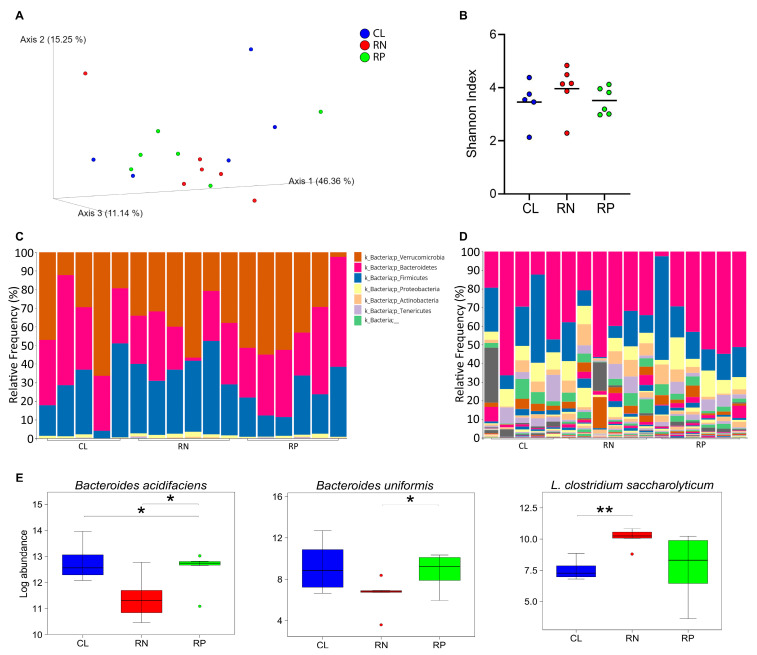
Reducing amino acid content of diet induces a modest shift in bacterial populations. 16S rRNA profiling of bacteria was carried out on feces from mice fed control (CL), reduced protein (RP) or reduced NEAA (RN) diets for two weeks. (**A**) Data are shown as principal coordinate analysis. (**B**) Diversity of bacteria type, as determined by Shannon Index, is shown for each group. Black line indicates average value for each group. (**C**,**D**) Relative abundance of bacteria in each group is shown at the phyla (**C**) and species levels (**D**). (**E**) Those species that were higher than 1% of overall abundance in any one group and significantly different in at least one group are shown as log abundance. * *p* < 0.05; ** *p* < 0.01.

Because bacteria are major contributors to the metabolome of the host, and diet is a major factor in modifying bacterial populations in the gut [26,27,28,29,30], we next tested whether the metabolomic changes induced by reducing dietary protein or NEAAs were associated with alterations in bacteria. To test this, 16S rRNA profiling was conducted on fecal samples collected from mice fed CL, RP or RN diets for two weeks. As shown in Figure 5A,B, principal coordinate analysis (PCoA) revealed no clear separation of groups, nor was diversity appreciably different. Deeper analysis of the percent abundance of bacteria did not show any differences across phyla; however, changes were observed at the species level (Figure 5C,D, Figure S3 and Supplementary Table S2). Specifically, of those bacteria that made up over 1% of the overall abundance in any one group, *Bacteroides acidifaciens* and *Bacteroides uniformis* were found to be decreased in feces from mice fed the RN diet, compared to the CL and RP diets, respectively (Figure 5E and Supplementary Table S2). Additionally, *Lachnoclostridium clostridium saccharolyticum* was increased in the RN group, when compared to CL diet-fed mice (Figure 5E and Supplementary Table S2). Additional changes were found for several species making up less than 1% of the total abundance in any one group in mice fed either diet (Supplementary Table S2 and Supplementary Figure S3). Collectively, although modifying amino acid intake does not exert a strong overall effect on gut bacterial populations, select species are impacted.

## 4. Discussion

The use of metabolomics to inform on diet-induced changes in physiologic sites is growing, but further insight into how to utilize this technology and its accompanying computational analyses is needed. This study describes a comprehensive profiling of the metabolomes of the liver, serum and feces from mice fed a diet reduced in total protein or NEAAs, along with determination of microbial abundance. Here, we found that reducing NEAAs induced a more profound effect on the metabolite profiles in all three depots compared to reducing total protein, with the greatest number of changes found in the liver compared to serum or feces. Selective analysis of amino acids revealed a more robust impact of the RN diet on both NEAAs and EAAs, compared to the reduced protein diet. Lastly, although neither experimental diet induced a major shift in the overall microbiota profile, select species changed in abundance, mainly in those mice receiving the reduced NEAA diet.

Protein and protein-derived amino acids have emerged as key mediators of diseases, including cancer, and as such, there is strong interest in depriving these nutrients (potentially through diet) to prevent or treat pathologies [6,31,32,33]. Therefore, having a comprehensive understanding of the physiologic impact of modifying their availability is critical. Our analyses focused on serum showed that only one NEAA decreased upon feeding the RP diet, while four decreased in the RN diet. Of note, this difference in impact on this class of amino acids between the experimental diets occurred despite having equivalent reductions in total NEAA levels across diets. Further, an equivalent number of EAAs decreased in both experimental groups despite no reduction in EAA concentration in the reduced NEAA diet. These findings are consistent with a previous study switching pigs from a control diet to a low protein diet (thus reducing intake by ~25%) [34]. Here, the authors found that although few changes in the levels of plasma NEAAs were observed, a number of EAAs decreased [34]. These results highlight the fact that alterations in dietary constituents may not always directly result in identical changes in circulation, but instead, corresponding physiologic responses to the dietary change could impact circulating metabolite levels. Moreover, it is possible that the differences in the formulation of either diet in the current study impact metabolism and absorption of their constituents, given that total protein in the form of casein was decreased in the RP diet while individual essential amino acids were added back after lowering total protein content in the RN diet. It should be noted that dietary intervention trials carried out in humans subjects by our group and others demonstrate that changes in circulating branched chain amino acids as a class corresponded with the amount of total protein consumed [35,36], an effect that was largely recapitulated in the current study using mice. Therefore, regardless of the mechanism by which these AAs change, branched chain amino acids could be considered biomarkers of protein consumption to help inform on adherence to dietary interventions in clinical trials.

The liver is acutely sensitive to alterations in diet, including protein, and as such, plays a major role in physiologic responses to such changes [37,38,39,40]. In line with this, we found the greatest number of metabolomic changes in this matrix compared to serum or feces in mice fed either experimental diet, including alterations in AAs. Interestingly, although EAAs were not lower in the RN diet, compared to the control group, a number of AAs in this class changed in the liver. It is possible that such changes reflect utilization of EAAs when NEAAs are not as abundant, which may be unsurprising given the responsiveness of the liver to modulation of NEAAs. It should be noted that although typical pathways that are upregulated in the liver in response to reduced availability of protein/amino acids (i.e., eIF2, GCN2, etc.) in an attempt to reduce protein translation were not measured in the current study, it is likely that these mediators were altered, based on previous work [40]. Another consideration is whether metabolite changes in the liver can impact circulation or vice versa, especially within AAs. Our data show that although several AAs had parallel changes in both matrices, many alterations were distinct, only occurring in either site. It is possible that changes that occurred in only one sample type reflect differences in the paths of digestion, absorption and/or utilization of nutrients.

Work from our group and others has shown gut microbiota to be an important contributor to both the intestinal and circulating metabolome given their ability to both utilize and secrete small molecules [26,29,30]. Given the impact diet can have on microbes, it is reasonable to posit that metabolomic shifts induced by dietary alterations could, in part, occur through gut microbiota. The results described in the current study revealed that reducing dietary amino acids exerted the fewest metabolomic changes in feces compared to the other two matrices analyzed. Although, it should be noted that the platform used in this study was restricted to polar metabolites and therefore did not take into account potential changes in bile acids, lipids, short-chain fatty acids and other more microbial-specific molecules. Besides diet-induced alterations in microbial metabolism, changes in nutrient availability are capable of shifting the abundance of microbial populations [27,28]. In fact, changes in the relative abundance of select species were observed upon reducing amino acid intake of mice; although, overall, the impact of the experimental diets on bacterial populations was modest. More in-depth analysis of microbial gene abundance or expression will be required to confidently link the microbial and metabolomic changes observed here.

Although we believe these findings have important implications for understanding physiologic responses to dietary modifications, some limitations of the study should be noted. A limited number of mice were used to examine the reported diet-mediated changes, highlighting the need to confirm these findings in a larger cohort in future studies. Additionally, given that this study increased dietary fat (in the form of soybean oil) to make the diets isocaloric following reductions in protein/AAs, we cannot exclude the possibility that this elevation in lipids could mediate some of the observed changes. Certainly, future studies are warranted to determine whether similar metabolomic and microbial changes occur when carbohydrates are used to replace calories lost by lowering protein/AAs.

## 5. Conclusions

Findings from this work lead to the conclusions that dietary protein and AAs are able to modulate gut, circulating and liver metabolites. Further, these dietary factors are capable of impacting intestinal microbial populations. Importantly, given recent interest in modifying nutrient intake for altering the pathogenesis of disease in patients, including cancer, these findings provide insight into potential physiologic effects that may occur in the context of such an approach. Although our work demonstrates that, at least with reduced amino acid intake, no obvious detrimental effects occurred, future studies in humans using a similar approach or other modifications should be sufficiently monitored to ensure safety.

## Figures and Tables

**Table 1:** Composition of experimental diets.

	Control		Reduced Protein		Reduced NEAA	
Ingredient	gm		gm		gm	
Casein	200		100		100	
L-Histidine	0		0		2.25	
L-Isoleucine	0		0		3.75	
L-Leucine	0		0		7.85	
L-Lysine	0		0		6.5	
L-Methionine	0		0		2.5	
L-Phenylalanine	0		0		4.15	
L-Threonine	0		0		3.55	
L-Tryptophan	0		0		1.05	
L-Valine	0		0		4.6	
L-Cystine	3		1.5		1.5	
Corn Starch	397.5		397.5		397.5	
Maltodextrin 10	132.0		132.0		132.0	
Sucrose	107.1		107.1		107.1	
Cellulose	50.0		50.0		50.0	
Soybean Oil	70.0		109.3		93.7	
t-Butylhydroquinone	0.01		0.01		0.01	
Mineral Mix S10022C	3.5		3.5		3.5	
Calcium Carbonate	12.5		9.5		10.0	
Calcium Phosphate, Dibasic	0.0		3.0		3.5	
Potassium Citrate, 1 H20	2.5		2.3		2.5	
Potassium Phosphate, Monobasic	6.9		6.5		6.9	
Sodium Chloride	2.6		2.6		2.6	
Vitamin Mix V10037	10.0		10.0		10.0	
Choline Bitrartrate	2.5		2.5		2.5	
Total	1000.0		937.3		959.4	
	gm	kcal	gm	kcal	gm	kcal
Protein	179.0	716.0	89.5	358.0	125.7	502.8
Carbohydrate	646.6	2586.3	646.6	2586.3	646.6	2586.3
Fat	70.0	630.0	109.3	983.7	93.7	843.3
Fiber	50.0	0.0	50.0	0.0	50.0	0.0
Total		3932.3		3928.0		3932.4
kcal/gm	3.9		4.2		4.1	
	gm%	kcal%	gm%	kcal%	gm%	kcal%
Protein	17.9	18.2	9.5	9.1	13.1	12.8
Carbohydrate	64.7	65.8	69.0	65.8	67.4	65.8
Fat	7.0	16.0	11.7	25.0	9.8	21.4

## Data Availability

The original contributions presented in the study are included in the article/Supplementary Material; further inquiries can be directed to the corresponding authors. Analysis code is available on GitHub at https://github.com/wcmq-abmc/mice_dietary_study/tree/master.

## Supplementary Materials

The following supporting information can be downloaded at https://www.mdpi.com/article/10.3390/metabo14120706/s1. Figure S1: Body weight and calorie intake are similar across study diets; Figure S2: Reducing amino acid intake alters the abundance of metabolites involved in glycolysis the TCA cycle; Figure S3: Abundance of all microbial species following dietary modulation of amino acids; Table S1: Summary statistics of all associations; Table S2: Relative abundance of bacterial species.
