# Supplementary material for: A Comprehensive Metabolomic and Microbial Analysis Following Dietary Amino Acid Reduction in Mice
Source: Metabolites. 2024 Dec 14;14(12):706. doi: 10.3390/metabo14120706 (PMC11677231; doi:10.3390/metabo14120706)

SUPPLEMENTARY FIGURES

Supplementary Figure S1. Body weight and calorie intake are similar across study diets. Mice were pair-fed isocaloric diets control (CL), reduced protein (RP) or reduced NEAA (RN) diets for two weeks. Body weight changes (relative to the initiation of diets) (A) and calorie intake (B) were calculated.

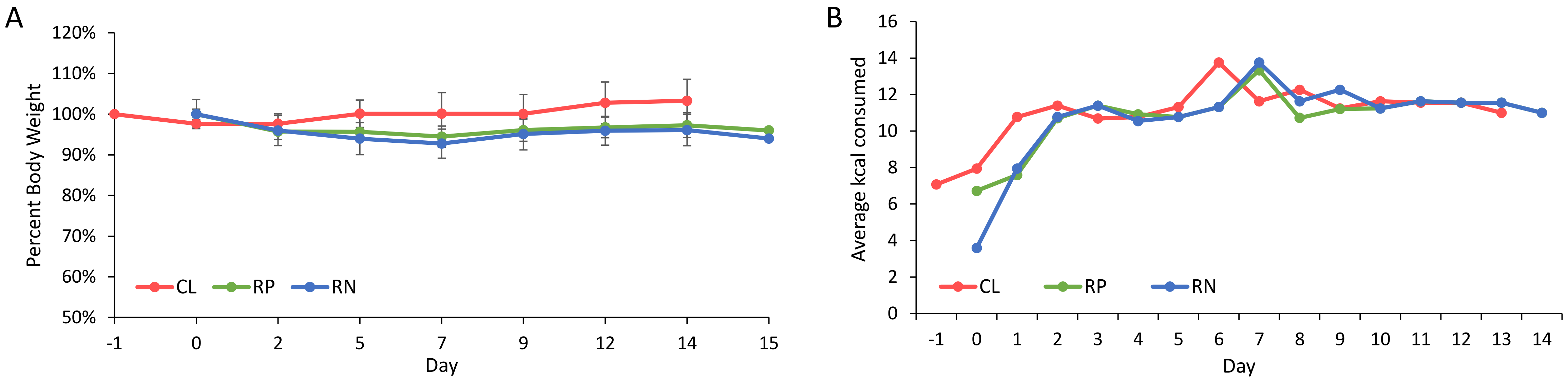

Supplementary Figure S1

Supplementary Figure S2. Reducing amino acid intake alters the abundance of metabolites involved in glycolysis the TCA cycle. A-B) The relative levels of metabolites in glycolysis (A) and the TCA cycle (B) were measured in the livers of mice fed a reduced protein (RP) or reduced NEAA (RN) diet compared to mice given a control (CL) diet. *P<0.05; **P<0.01; P<0.001.

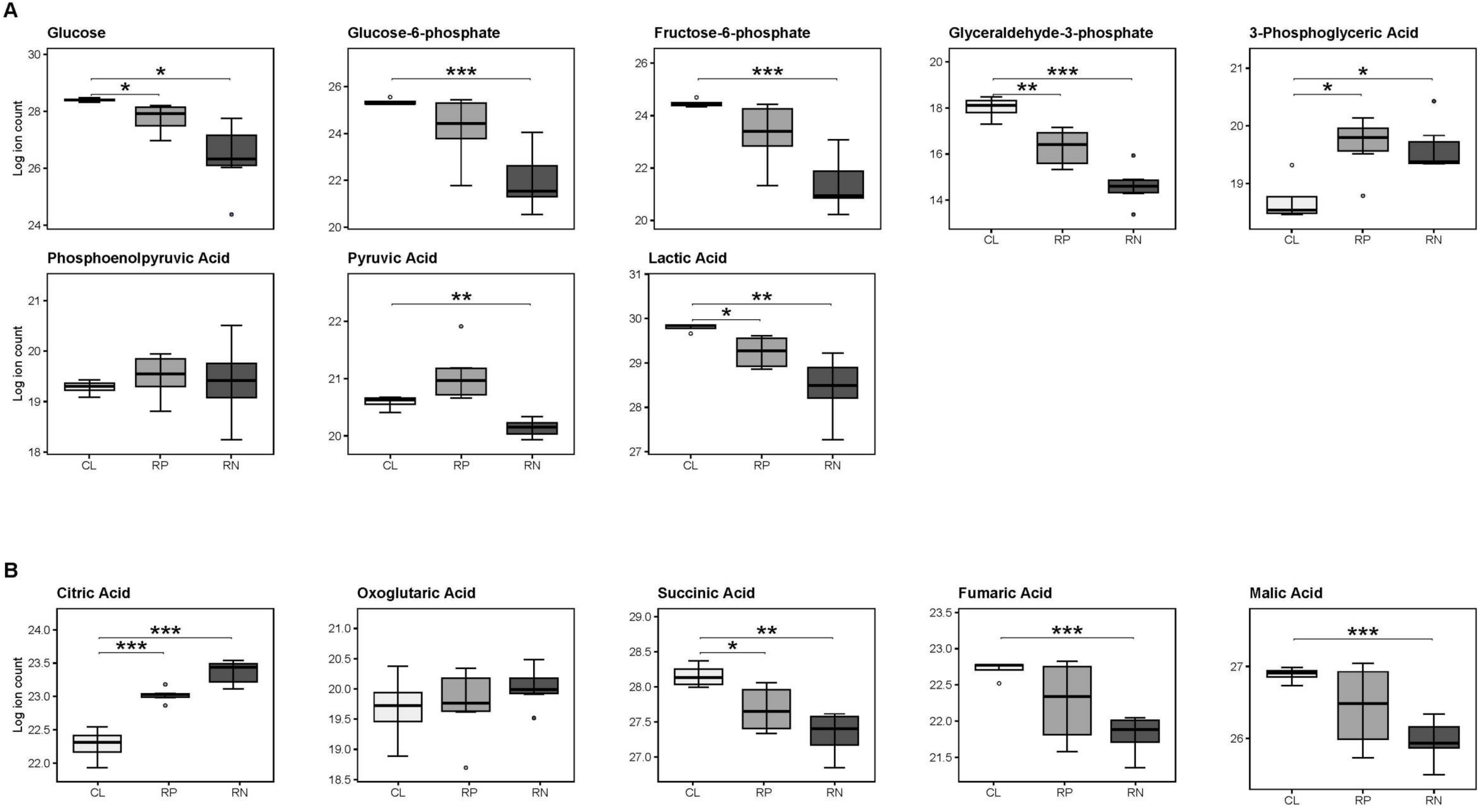

Supplementary Figure S2

Supplementary Figure S3. Abundance of all microbial species following dietary modulation of amino acids. Log abundance of bacterial species in feces from mice fed control (CL), reduced protein (RP) or reduced NEAA (RN) diets, are shown. *P<0.05; **P<0.01; P<0.001.

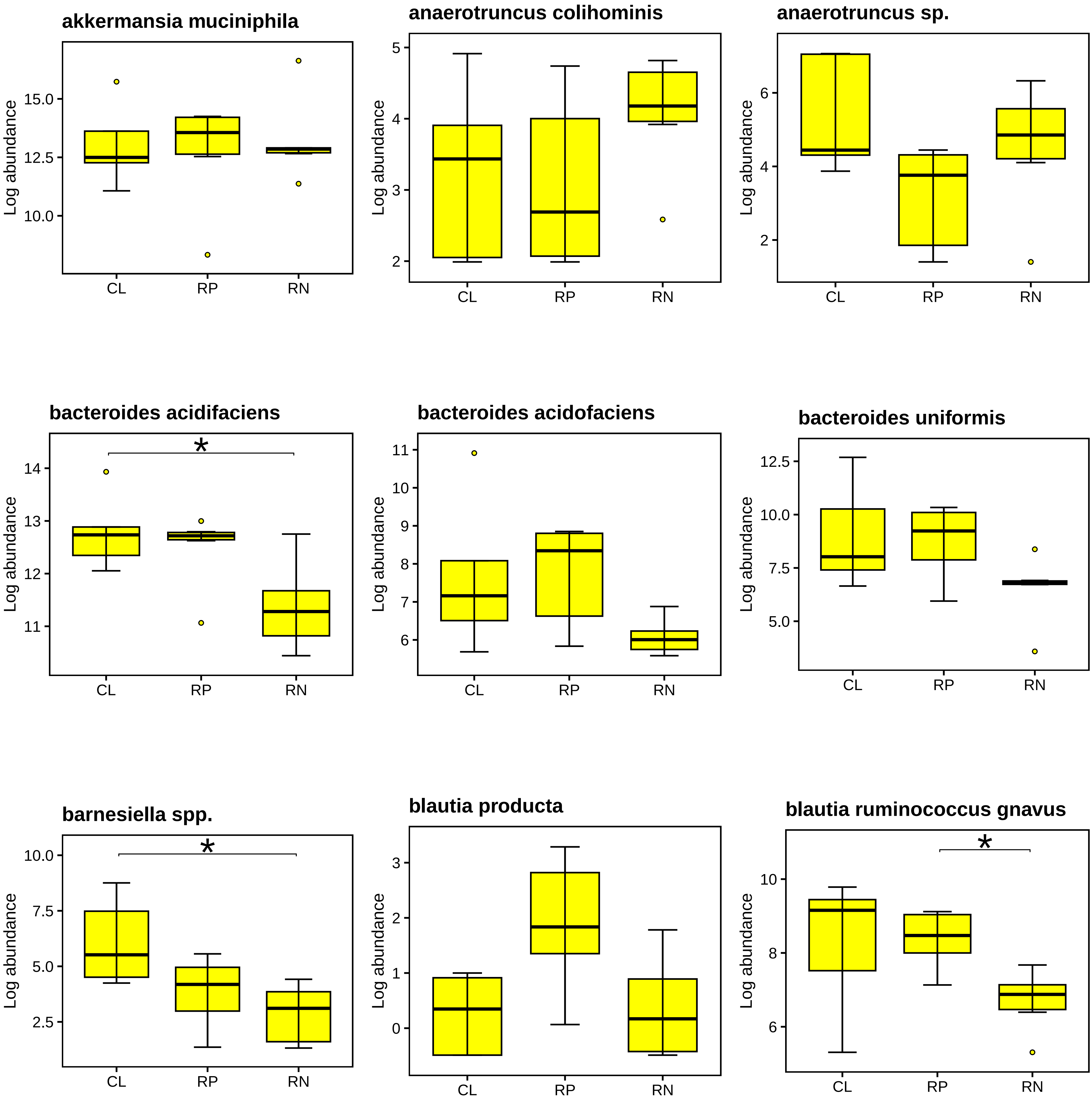
akkermansia muciniphila
anaerotruncus colihominis
anaerotruncus sp.
bacteroides acidifaciens
bacteroides acidofaciens
bacteroides uniformis
barnesiella spp.
blautia producta
blautia ruminococcus gnavus
Log abundance
CL
RP
RN
*

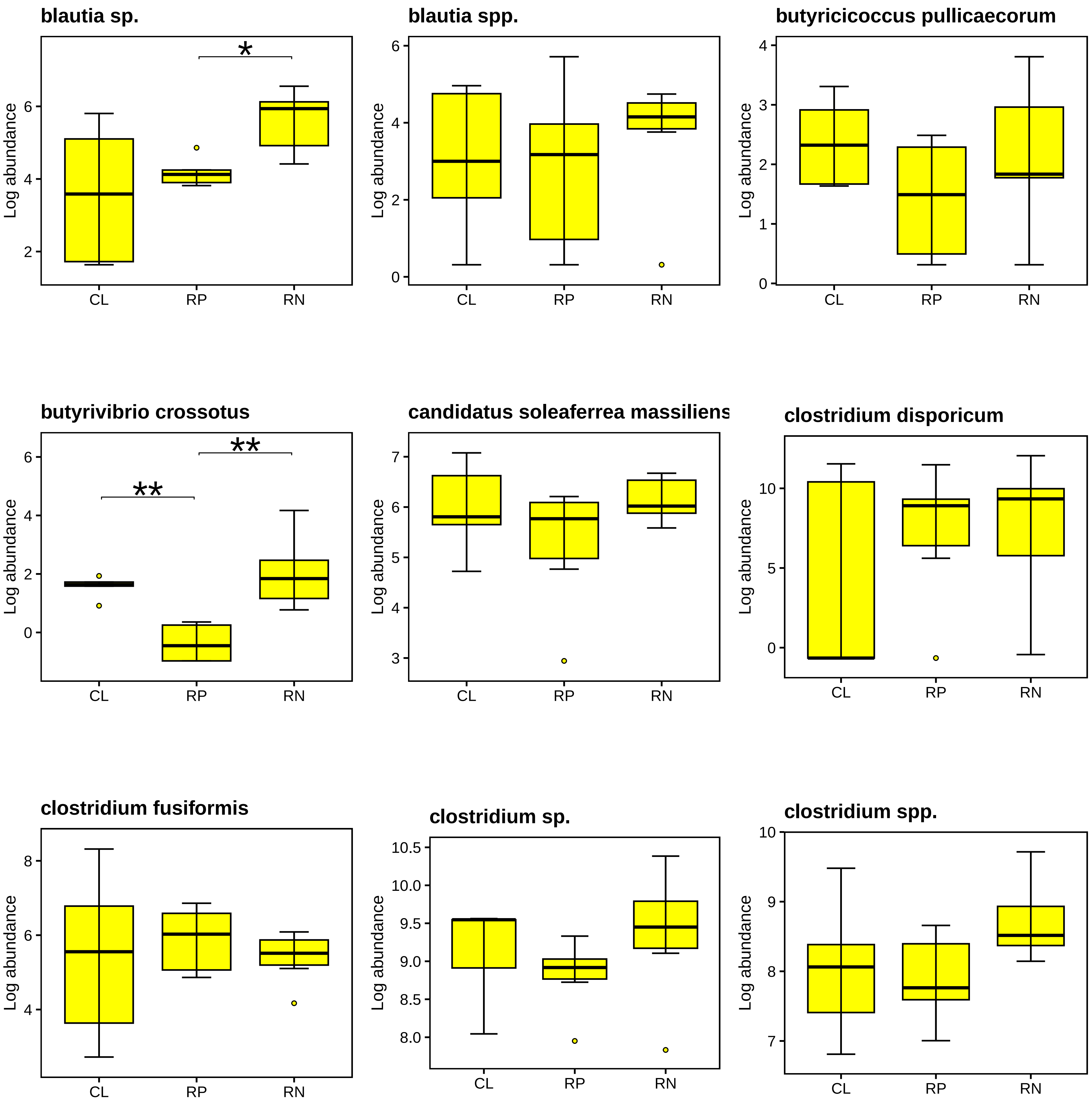
blautia sp.
*
blautia spp.
butyricicoccus pullicaecorum
butyrivibrio crossotus
**
**
candidatus soleaferrea massiliens
clostridium disporicum
clostridium fusiformis
clostridium sp.
clostridium spp.
Log abundance
CL
RP
RN

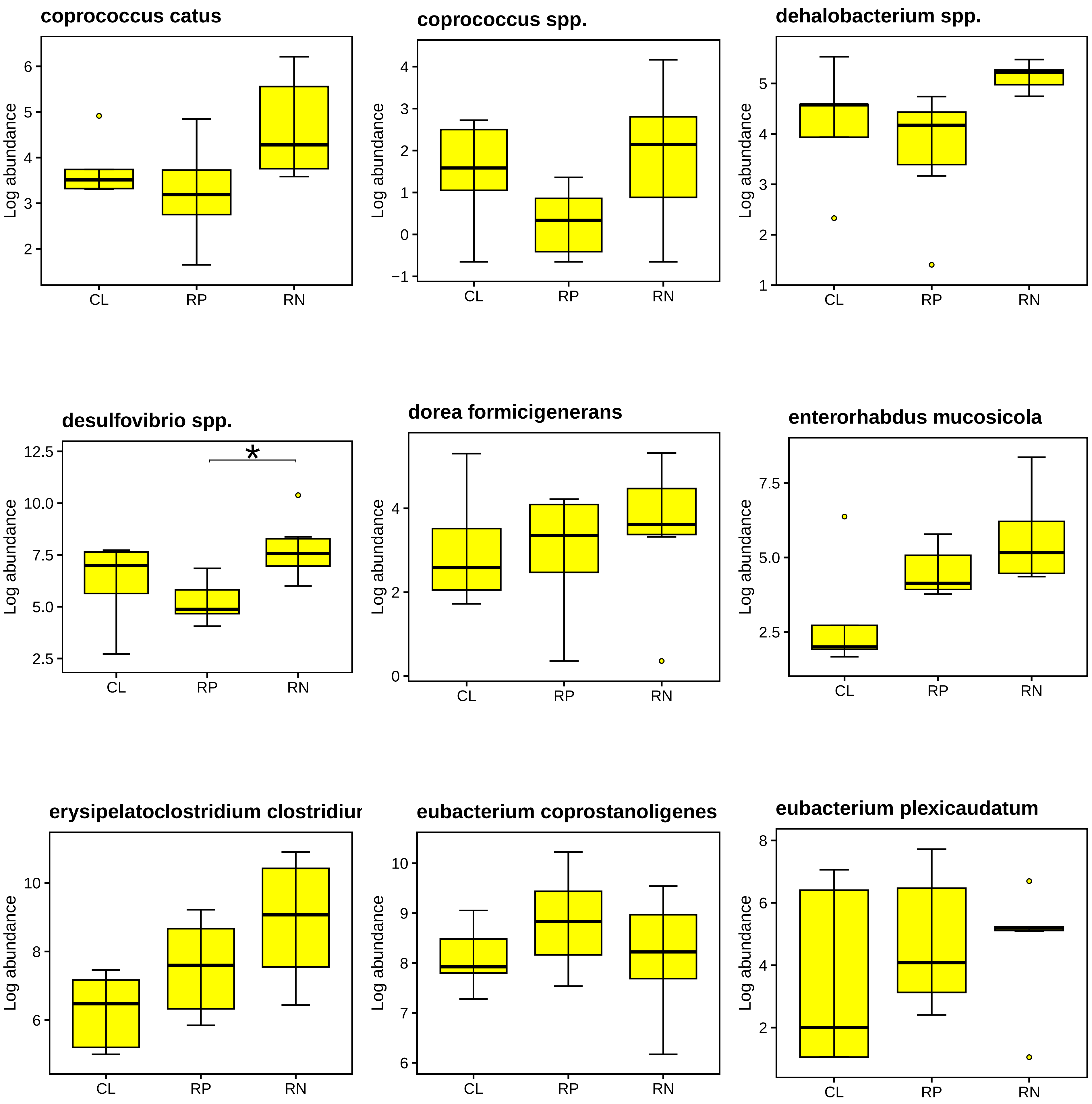
coprococcus catus
Log abundance
6 5 4 3 2
CL RP RN
coprococcus spp.
Log abundance
4 3 2 1 0 −1
CL RP RN
dehalobacterium spp.
Log abundance
5 4 3 2 1
CL RP RN
desulfovibrio spp.
*
Log abundance
12.5 10.0 7.5 5.0 2.5
CL RP RN
dorea formicigenerans
Log abundance
4 2 0
CL RP RN
enterorhabdus mucosicola
Log abundance
7.5 5.0 2.5
CL RP RN
erysipelatoclostridium clostridiur
Log abundance
10 8 6
CL RP RN
eubacterium coprostanoligenes
Log abundance
10 9 8 7 6
CL RP RN
eubacterium plexicaudatum
Log abundance
8 6 4 2
CL RP RN

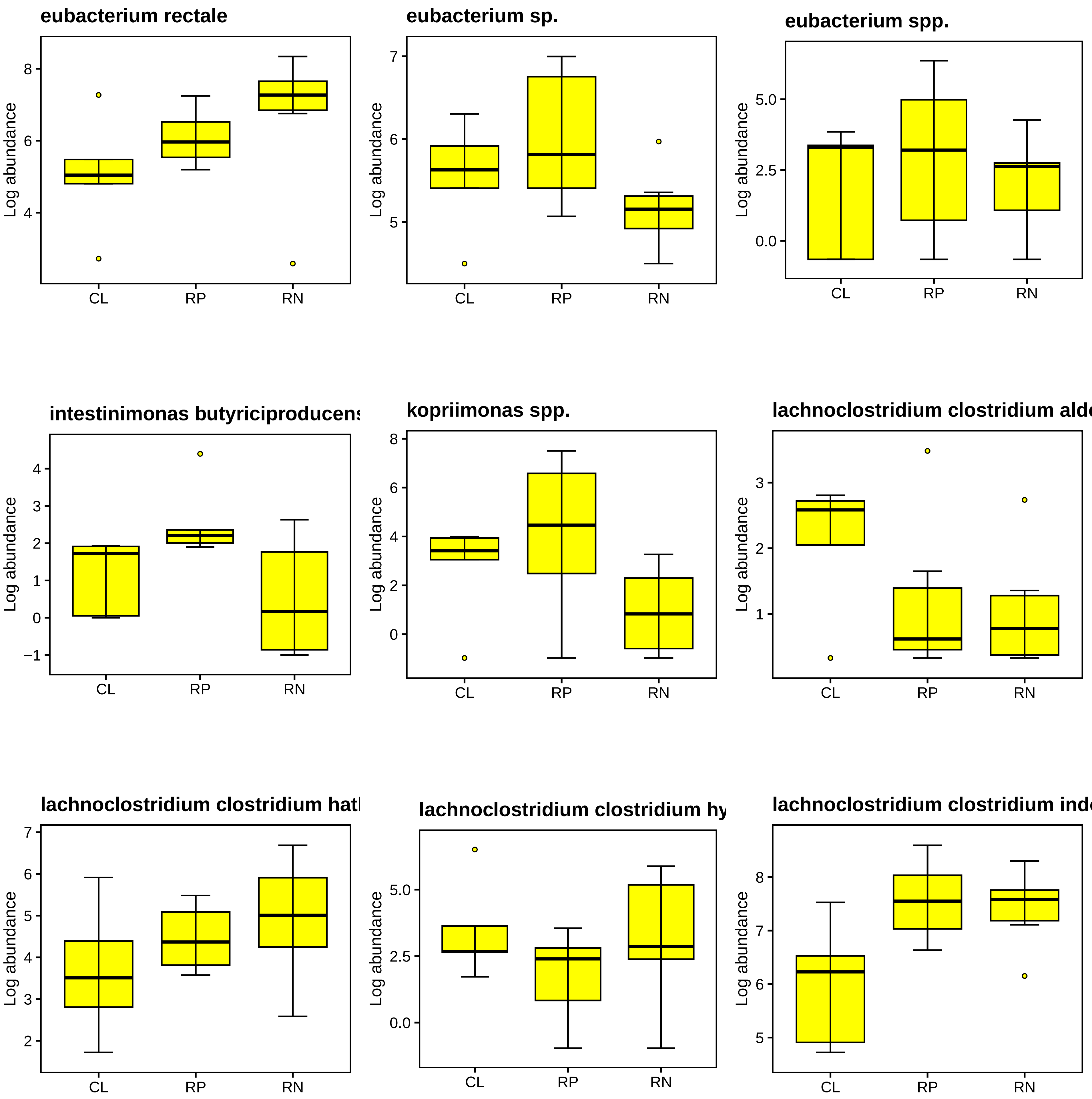
eubacterium rectale
Log abundance
CL RP RN
eubacterium sp.
Log abundance
CL RP RN
eubacterium spp.
Log abundance
CL RP RN
intestinimonas butyriciproducens
Log abundance
CL RP RN
kopriimonas spp.
Log abundance
CL RP RN
lachnoclostridium clostridium aldo
Log abundance
CL RP RN
lachnoclostridium clostridium hatl
Log abundance
CL RP RN
lachnoclostridium clostridium hy
Log abundance
CL RP RN
lachnoclostridium clostridium indo
Log abundance
CL RP RN

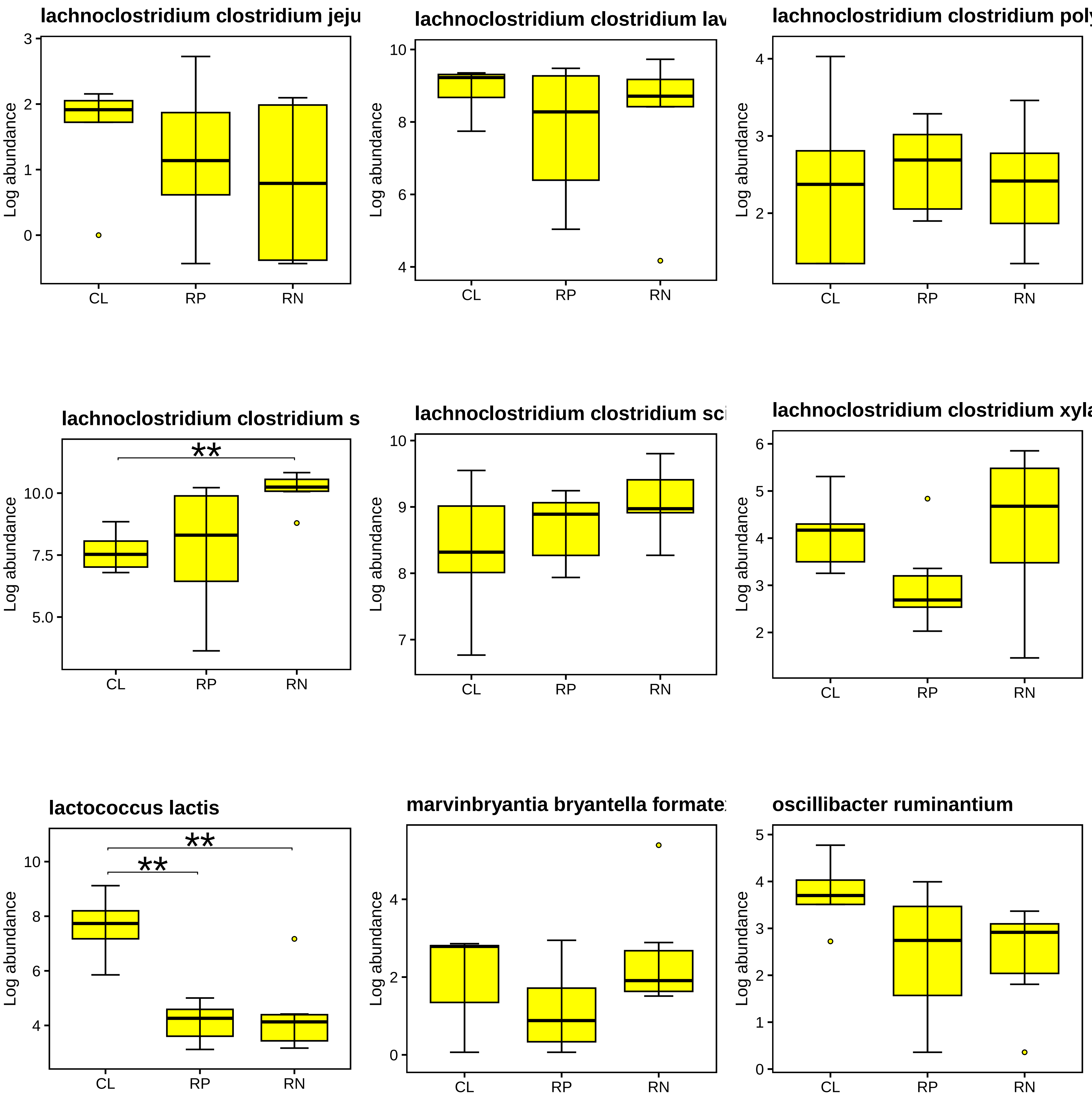
lachnoclostridium clostridium jeju
lachnoclostridium clostridium lav
lachnoclostridium clostridium poly
lachnoclostridium clostridium s
lachnoclostridium clostridium sci
lachnoclostridium clostridium xyla
lactococcus lactis
marvinbryantia bryantella formate
oscillibacter ruminantium
Log abundance
CL
RP
RN
**
**
**

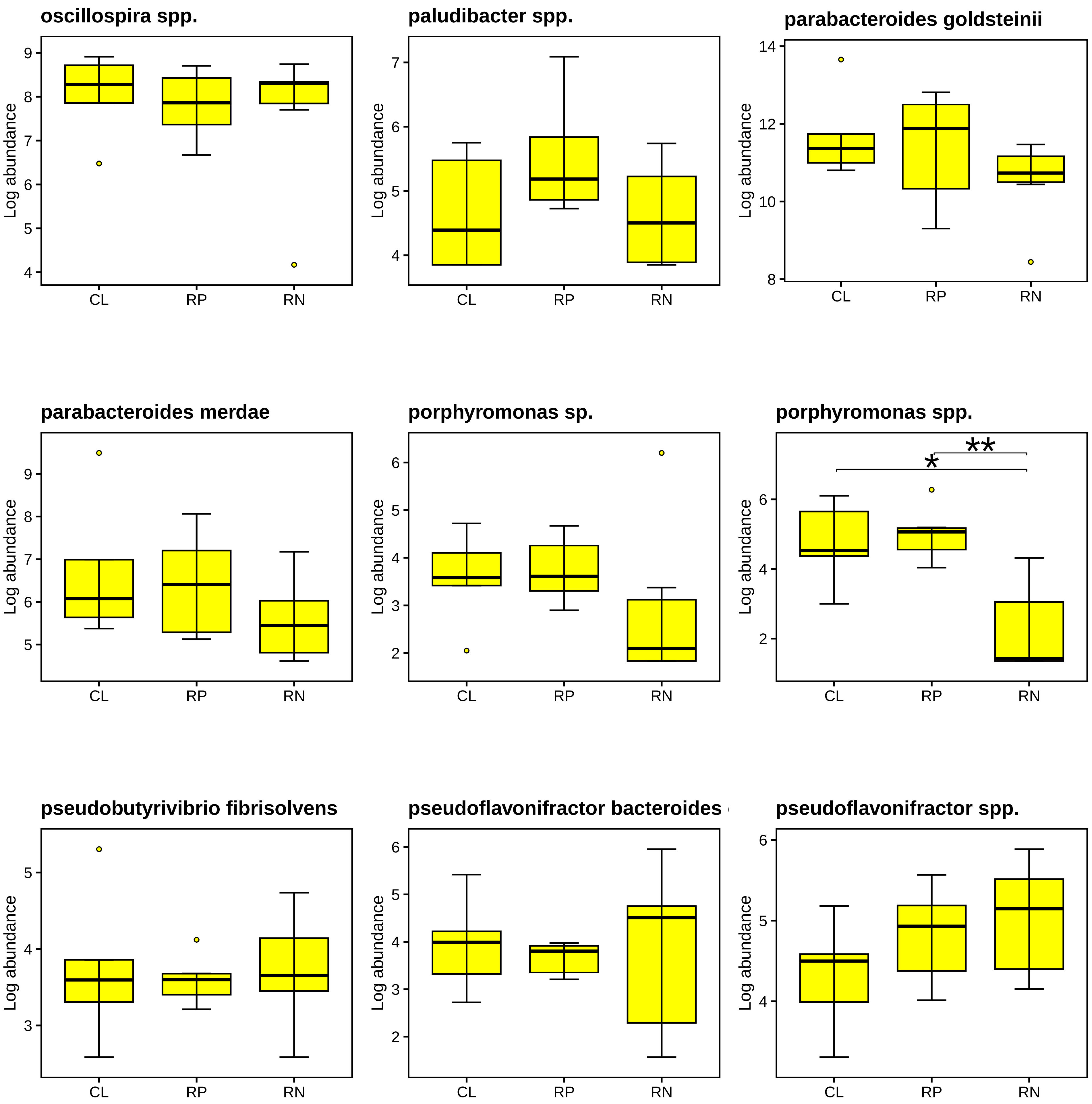

oscillospira spp.
paludibacter spp.
parabacteroides goldsteinii
parabacteroides merdae
porphyromonas sp.
porphyromonas spp.
**
*
pseudobutyrivibrio fibrisolvens
pseudoflavonifractor bacteroides
pseudoflavonifractor spp.
Log abundance
CL
RP
RN

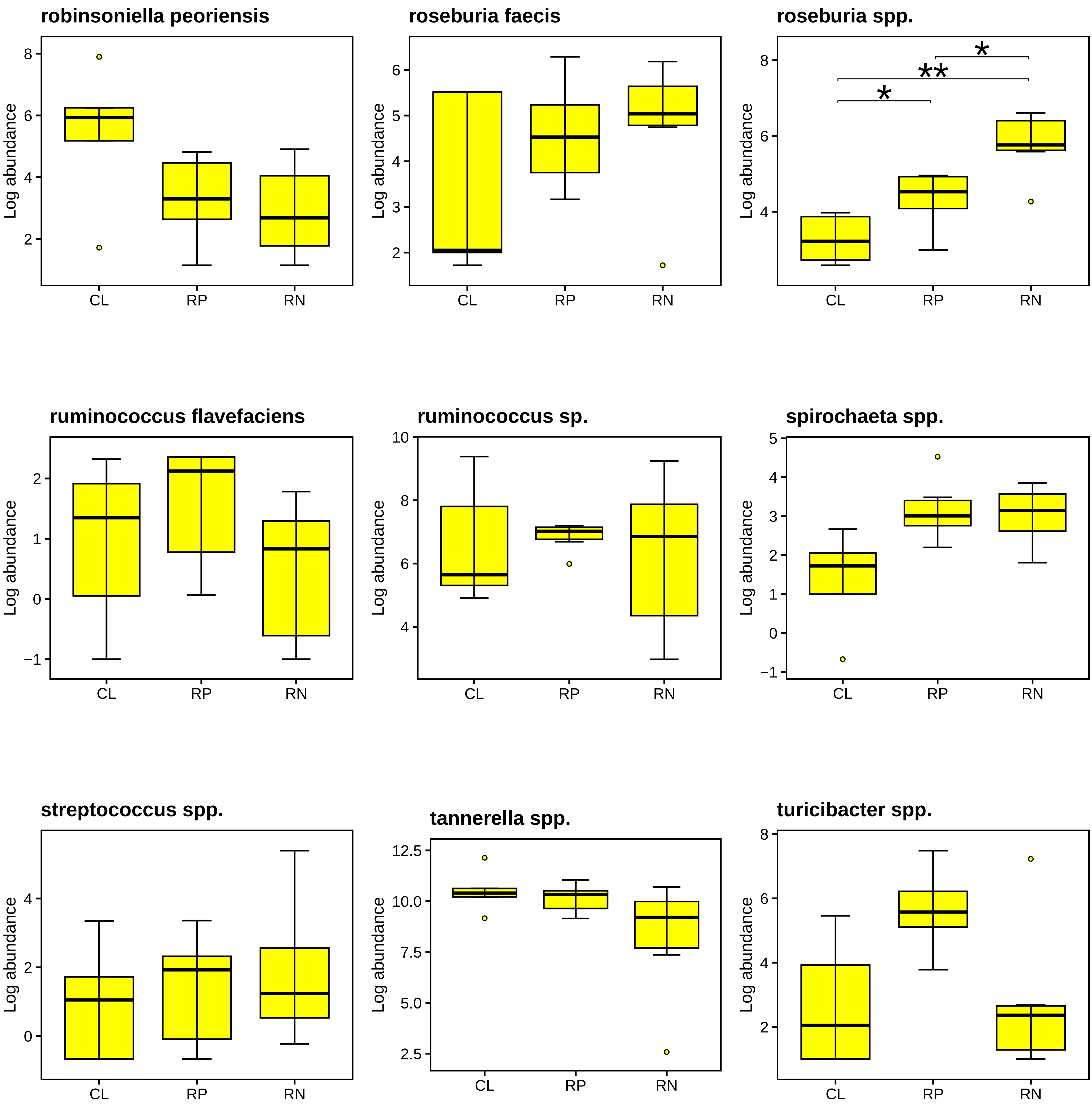
robinsoniella peoriensis
Log abundance
8
6
4
2
CL
RP
RN
roseburia faecis
Log abundance
6
5
4
3
2
CL
RP
RN
roseburia spp.
Log abundance
8
6
4
*
**
*
CL
RP
RN
ruminococcus flavefaciens
Log abundance
2
1
0
−1
CL
RP
RN
ruminococcus sp.
Log abundance
10
8
6
4
CL
RP
RN
spirochaeta spp.
Log abundance
5
4
3
2
1
0
−1
CL
RP
RN
streptococcus spp.
Log abundance
4
2
0
CL
RP
RN
tannerella spp.
Log abundance
12.5
10.0
7.5
5.0
2.5
CL
RP
RN
turicibacter spp.
Log abundance
8
6
4
2
CL
RP
RN

tyzzerella clostridium lactatifermentans

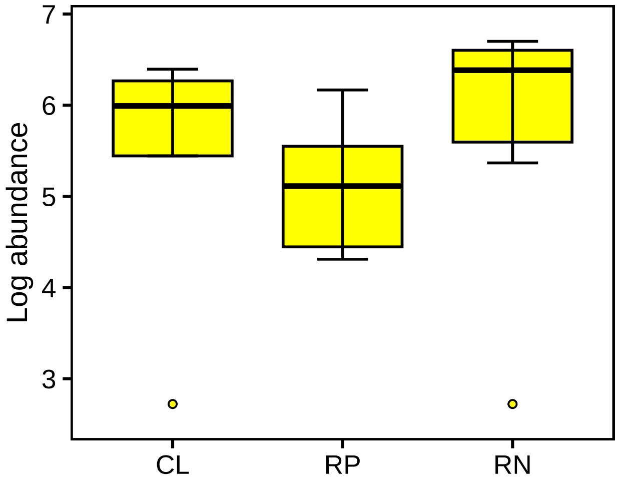

Supplement: Supplementary file 1 [file metabolites-14-00706-s001.zip › Supplementary Figures.pdf]
